# Spontaneous Ruptured Pyomyoma in a Nulligravid Female: A Case Report and Review of the Literature

**DOI:** 10.1155/2018/1026287

**Published:** 2018-07-02

**Authors:** S. Read, J. Mullins

**Affiliations:** ^1^UConn Health, Department of OB/GYN, Farmington, CT, USA; ^2^Hartford Hospital, Department of OB/GYN, Hartford, CT, USA

## Abstract

**Introduction:**

Pyomyoma, or suppurative leiomyoma, is a rare complication of uterine fibroids. It occurs most commonly in the setting of pregnancy, the immediate postpartum period, or postmenopausal status. It may also arise after recent uterine instrumentation, after uterine artery embolization, or in immunocompromised patients. The most likely cause of pyomyoma is vascular compromise followed by bacterial seeding from direct, hematogenous, or lymphatic spread. Diagnosis is difficult, as the condition is rare, presents with vague symptoms, and is difficult to identify on imaging. Definitive diagnosis is only possible with surgery. Pathology shows a degenerating fibroid with hemorrhage, necrosis, cystic degeneration, and/or inflammatory change. Cultures of the pus contained within often show polymicrobial infection.

**Case Presentation:**

Our patient is a 24-year-old nulligravid female who presented with a surgical abdomen, fever, hypotension, and leukocytosis. She had no significant prior medical or surgical history, no history of uterine instrumentation, and no history of pelvic infection; she was not currently sexually active at the time of presentation. She was taken to the operating room, where she underwent diagnostic laparoscopy. This showed a ruptured pyomyoma originating in the left broad ligament. She then underwent laparoscopic myomectomy. She was transferred to the ICU intubated; she slowly recovered on IV antibiotics and was discharged home on postoperative day 10.

**Discussion:**

Pyomyoma is a rare condition and is even rarer in premenopausal patients without recent history of pregnancy or uterine instrumentation. This demonstrates an unusual case of spontaneous pyomyoma in the absence of risk factors, other than a history of known fibroids. Pyomyoma should be considered as a diagnosis in patients with sepsis, history of fibroids, and no other identifiable source of infection.

## 1. Introduction

Leiomyomata are a common benign smooth muscle neoplasm in women, occurring in 20-30% of premenopausal women [1]. The cumulative incidence is approximately 70% in Caucasian women and may be as high as 80% in Afro-Caribbean women [2]. Pyomyoma, or suppurative leiomyoma, is a rare condition that occurs with infection of a leiomyoma. The infected leiomyoma generally shows suppurative inflammation, containing pus with neutrophils and necrotic exudate [3]. Most cases in premenopausal women occur during pregnancy or the immediate postpartum period, after uterine instrumentation [3], or as a result of cervical stenosis [4]. Only four cases have been reported as being diagnosed during pregnancy [5]. Cases in postmenopausal women are likely the result of immune or vascular compromise, such as in the setting of diabetes, hypertension, or atherosclerotic disease [3]. Infections are generally polymicrobial and are thought to arise from the lower genital tract, by direct spread—for example, by contact with the endometrial cavity in a C-section [6]—or by hematogenous/lymphatic spread [1, 3, 7]. Cases in the immediate postpartum period or after uterine instrumentation, e.g., after a D&C, are thought to be most likely the result of ascending infection [1]. Use of instrumentation in the case of postpartum hemorrhage, including intrauterine balloons, also increases risk of infection, while hemorrhage increases risk of infarction of fibroids [8]. Recently, uterine artery embolization (UAE) has become more popular as a treatment for fibroids, as it offers an alternative to traditional surgical and medical therapies [3, 9]. After its introduction in 1996, there have been multiple case reports of pyomyoma forming after UAE [9–12]. This is thought to be a result of uterine and leiomyomatous ischemia [9]. The mechanism of pyomyoma formation is most likely infarction followed by necrosis, with subsequent infection and pus formation [9].

Diagnosis of pyomyoma is difficult, as it is a relatively rare condition and may develop over an extended period of time [4]. Patients typically present with abdominal pain and fever; the triad that should raise suspicion is sepsis, history of fibroids, and absence of another source of infection [1, 9]. However, the presentation in postmenopausal women may be similar to malignancy, with symptoms of abdominal distension/bloating, anorexia, and change in stools [13]. CA-125 may also be elevated [14]. Imaging is often nonspecific; diagnosis cannot truly be made until the time of surgery [14]. Ultrasound shows heterogeneous uterine masses, sometimes with cystic components or air [1, 3]. Abulafia et al. (2010) proposed that an anechoic halo of normal myometrium surrounding the mass may be highly suggestive or even pathognomonic for pyomyoma on ultrasound. CT shows a similar picture but may better demonstrate calcifications, free peritoneal air, and fluid with the density of purulent material [15]. MRI has not been shown to be helpful in diagnosis. The differential diagnosis includes pyometra, tuboovarian abscess, an infected ectopic pregnancy, malignancy, perforated viscus, or degenerating fibroids [2, 8]. The most feared complication of a pyomyoma is rupture, followed by sepsis and death [9]. Rupture is suggested on imaging by disruption of the wall of the pyomyoma, along with free intraperitoneal fluid and air [1]. Mortality rate estimates range from 6% to 21% in the era of antibiotics [5, 8, 14, 16]. Prior to the development of antibiotics in 1945, the mortality rate was 29% according to case series [17]. During pregnancy, occurrence of a pyomyoma may result in spontaneous abortion, preterm labor, preterm premature rupture of membranes (PPROM), or postpartum hemorrhage [17].

Due to the nonspecific presentation of pyomyoma, initiation of appropriate treatment is frequently delayed [16]. Treatment of pyomyoma is almost always surgical. It may be managed with hysterectomy or myomectomy, depending on the location of the pyomyoma and the severity of the patient's condition [1]. Perioperative antibiotics are also necessary; conservative treatment with antibiotics alone is generally ineffective [15]. On occasion, conservative management is possible with drainage and antibiotics. Laubach et al. (2011) presented a case series in which management was successfully performed with drainage by interventional radiology and IV antibiotic therapy in two of three cases. Chen et al. (2014) performed a literature review and found that in 48 cases, 32 underwent hysterectomy, 10 underwent myomectomy, and 6 underwent drainage. The patient's menopausal status and desire for future fertility should be taken into consideration when deciding which approach to take for management [15]. When possible, myomectomy alone is an important treatment option for women desiring maintenance of their fertility [16].

Pathology shows a degenerating fibroid containing hemorrhage, necrosis, or calcification [3]. The pyomyoma may demonstrate a “capsule” on imaging, which on pathology appears as a flattened layer of smooth muscle cells [3]. Cystic degeneration, hyaline change, and acute inflammatory change may also be present [18]. Cultures of the pus from the pyomyoma generally demonstrate polymicrobial infection; the most commonly isolated organisms are* Staphylococcus *species, but* Streptococcus *sp.,* E. coli, Pasteurella multocida*, and other organisms may also be identified [1, 3, 13, 19]. A case of* Candida*-related pyomyoma has also been described [20].

## 2. Case Presentation

Our patient is a 24-year-old nulligravid female with uncertain last menstrual period who presented to the emergency department (ED) with 12 hours of diffuse abdominal pain, worse in the left lower quadrant. She had intermittent nausea with one episode of emesis. She denied fevers or chills. She noted intermittent vaginal spotting, but no abnormal vaginal discharge. She had been seen two months previously for menorrhagia and was told at that time she had a possible fibroid; she was started on Depo-Provera for her menorrhagia. She denied any significant medical or surgical history. She denied any history of diabetes, HIV, or other immunocompromise. She denied any history of IUD placement or other uterine instrumentation. She had not been sexually active in several months and had no history of sexually transmitted infections.

**Figure 1:**
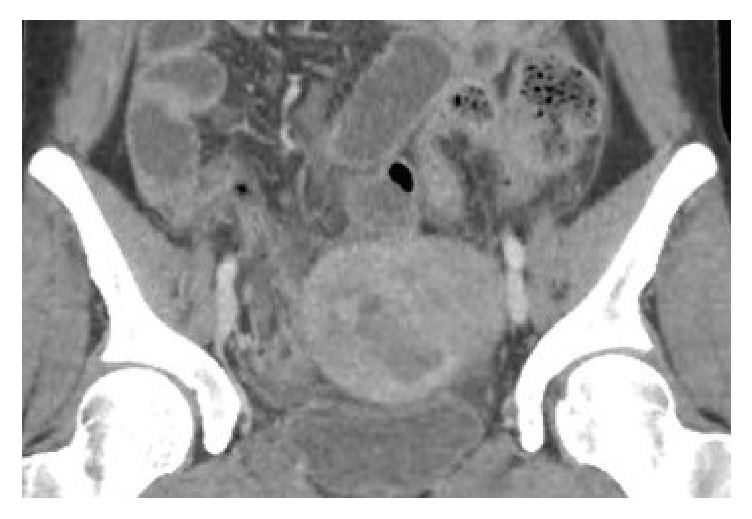
Heterogeneous pelvic mass on CT.

She was initially febrile to 100.9F in the ED; within a few hours her temperature increased to 103.6F, she became tachycardic to the 140s, and was hypotensive to the 80s/50s. Her WBC count was 17.8. Urine pregnancy and HIV tests were negative. Blood glucose was 164 on admission. She was started on IV fluids and pressors and was given doses of cefepime, ceftriaxone, doxycycline, and metronidazole. CT abdomen/pelvis with contrast showed an 8.1 x 5.5 x 5.6 cm heterogeneous mass in the deep left pelvis that was inseparable from the uterus and broad ligament; it had an incomplete solid ventral surface and was thought to represent a hemorrhagic or infarcted fibroid (Figure 1). No internal calcifications or fat was seen. Fat stranding and fluid were visible surrounding the mass. There was no pneumoperitoneum. Given the severity of the patient's condition and her hemodynamic instability, she was taken to the operating room for an exploratory laparoscopy.

**Figure 2:**
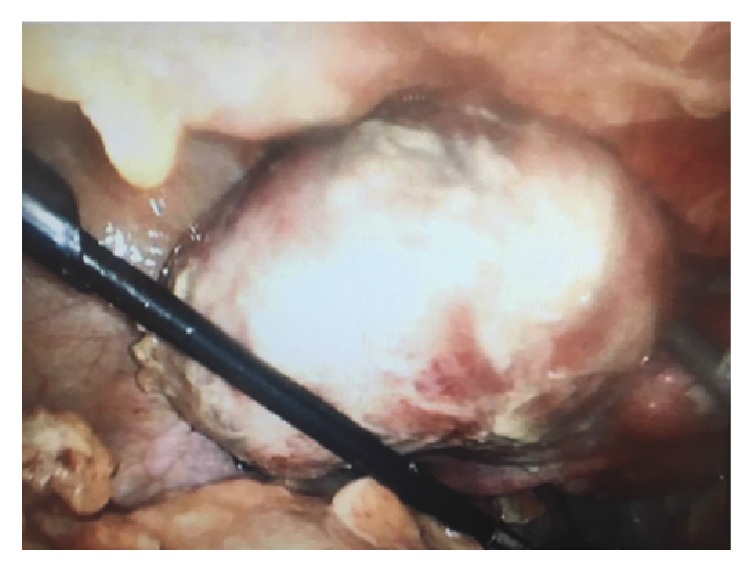
The left broad ligament pyomyoma. Purulent material is visible along the abdominal wall and bowel.

**Figure 3:**
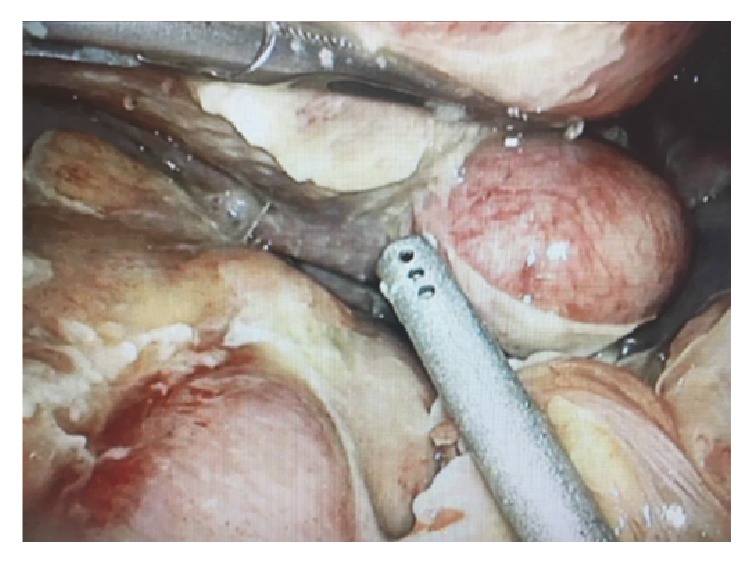
The uterine fundus is seen on the right hand side of the image. The pyomyoma is at the top of the image.

Intraoperatively, pus was noted throughout the abdomen and the patient's bowel was edematous and filled with gas. Multiple pus pockets were seen in the patient's pelvis. A large left broad ligament leiomyoma was noted and appeared to be leaking pus (Figures 2 and 3). A myomectomy was performed laparoscopically, and the patient's abdomen was washed out.

She was then taken to the ICU intubated on pressors. She was kept on tobramycin and clindamycin for a presumed tuboovarian abscess. She was extubated on postoperative day 2 but continued to require supplemental oxygen and pressors. She also continued to spike fevers to 101.3F and was persistently tachycardic to the 140s. Infectious disease recommended switching to ceftriaxone and metronidazole at that time. She was afebrile by postoperative day 4 and remained afebrile with the exception of one isolated elevated temperature on postoperative day 8. Her tachycardia resolved. Blood glucose remained within normal limits. She was transitioned to oral antibiotics and discharged home in good condition on postoperative day 10.

Pathology showed a 128-gram leiomyoma with partial ischemic changes and nonspecific inflammation. A gram stain of the pus showed many neutrophils and gram-negative rods; a culture of the purulent fluid showed no growth, indicating an anaerobic infection.

## 3. Discussion

Pyomyoma is a rare complication of leiomyomata and is generally seen in the context of pregnancy, postmenopausal status, or uterine instrumentation. This case is a rare exception, as the patient had no recent uterine instrumentation, was nulligravid, and had no reason to exhibit vascular compromise. No records were available from the time when her diagnosis of fibroids was made, so the size of her fibroid at the time of diagnosis is unknown. It is possible that she had rapid growth of the fibroid leading to ischemic changes, degeneration, and subsequent seeding with normal vaginal flora.

Pyomyoma should be suspected in cases of sepsis, history of fibroids, and no other clear source of infection, as was the case in this patient. For many patients found to have pyomyoma, symptoms may develop insidiously, and they may present with only a fever [21]. Imaging showed a heterogeneous pelvic mass, and culture of the pus from the pyomyoma showed no growth, indicating likely polymicrobial anaerobic infection. Surgical management was successfully performed laparoscopically; however, it would have also been reasonable to perform a laparotomy, which could have obtained a better washout. Luckily for this young, nulliparous patient, the pyomyoma was in her left broad ligament, and a fertility-sparing myomectomy was possible as a result. Fertility-sparing surgery should always be attempted if feasible, if the patient is premenopausal and desires future fertility, or if her childbearing wishes are unknown [15]. This patient did have a ruptured pyomyoma diagnosed at the time of surgery; luckily, she presented to the ED before she became hemodynamically unstable, and we were able to manage her appropriately.

In a review of 41 cases of pyomyoma diagnosed since 1986 (Table 1), 14 cases were treated with myomectomy (34%), 20 were treated with hysterectomy (49%), and the remainder were treated with IV antibiotics or minor procedures.

All patients received IV antibiotics during the course of their treatment. Of the 41 patients, only one patient died; she had been treated with IV antibiotics alone. The outcome of one of the patients was not reported. Since 2010, 10 of 25 cases were treated with hysterectomy (40%) [1–3, 5, 6, 8–19, 21–26], and only 2 of 11 patients under age 40 (18%) were treated with hysterectomy, so there may be a trend toward managing cases with conservative surgical treatments for fertility preservation purposes. The majority of cases were in premenopausal women or were associated with pregnancy, uterine artery embolization, or uterine instrumentation (e.g., IUD in place, D&E). Our patient is unique because of her young age, nulliparity, and lack of any prior uterine procedures or recent known infection. To our knowledge, this is the only known case to occur in a premenopausal woman without any history of uterine instrumentation or immunocompromise or in the peripartum period.

## Figures and Tables

**Table 1:** List of reviewed cases.

**Citation Number**	**Author**	**Age**	**Relevant History**	**Treatment**	**Outcome**
[3]	Ono	69	Postmenopausal	Hysterectomy	Unknown
[1]	Bagga	26	Post SAB	Myomectomy	Alive
[15]	Chen ZHY	46	Prior C-section (remote)	Hysterectomy	Alive
[9]	Obele	37	UAE	Hysterectomy	Alive
[18]	Goyal	42	Diabetic	Hysterectomy	Alive
[2]	Demaio	Unknown	Postpartum	Myomectomy	Alive
[14]	Iwahashi	53	IUD	Hysterectomy	Alive
[13]	Chen JR	69	Postmenopausal	Myomectomy	Alive
[16]	Pinton	28	Post SAB	Myomectomy	Alive
[8]	Kaler	28	Postpartum w/ PPH, intrauterine balloon	Myomectomy	Alive
[21]	Del Borgo	37	Postpartum	Myomectomy	Alive
[17]	Sirha	37	Postpartum	Hysterectomy	Alive
[6]	Shiota	36	C-section	Myomectomy	Alive
[10]	Rosen	47	UAE, untreated *Trichomonas* infection	Hysterectomy	Alive
[11]	Pinto	36	UAE	Drainage	Alive
[5]	Kobayashi	28	Pregnant	Myomectomy	Alive
[22]	Shaaban	30	Post C-section	Myomectomy	Alive
[23]	Liu	42	Myomectomy (remote)	Marsupialization	Alive
[24]	Stroumsa	41	D&E	IV antibiotics	Alive
[19]	Zangeneh	47	Perimenopausal; chronic endometritis on pathology	Hysterectomy	Alive
[25]	Laubach	31	D&E	Drainage	Alive
		35	C-section	Drainage	Alive
		31	C-section, surgical site infection	Drainage followed by hysterectomy	Alive
[12]	Abulafia	48	UAE	Hysterectomy	Alive
[26]	Lee	46	Unknown	Hysterectomy	Alive
[27]	Fletcher	44	Diabetic, PID	Hysterectomy	Alive
[28]	Nguyen	40	C-section, chorioamnionitis	Hysterectomy	Alive
[29]	Patwardhan	38	Torsion of pedunculated fibroid	Myomectomy	Alive
[30]	Manchana	42	Perimenopausal; IUD	Hysterectomy	Alive
[31]	Calleja-Agius	30	C-section	Myomectomy	Alive
[32]	Sah	64	Postmenopausal	Hysterectomy	Alive
[33]	Mason	29	Postpartum	Myomectomy	Alive
[4]	Karcaaltincaba	36	Post SAB	Myomectomy	Alive
[20]	Lin	33	C-section, surgical site infection	Hysterectomy	Alive
[34]	Grune	44	Pregnant	Myomectomy	Alive
[35]	Genta	60	Postmenopausal, diabetes	Hysterectomy	Alive
[36]	Gupta	75	Postmenopausal	Hysterectomy	Alive
[37]	Yang	46	Bacteremia	Hysterectomy	Alive
[38]	Prahlow	31	Pregnancy, IV drug use	Hysterectomy	Alive
[7]	Greenspoon	49	Bacteremia	IV antibiotics	Deceased
[39]	Pritchard	37	Post SAB, laparotomy	Hysterectomy	Alive

SAB: spontaneous abortion; UAE: uterine artery embolization; PID: pelvic inflammatory disease; D&E: dilation and evacuation; IUD: intrauterine device; PPH: postpartum hemorrhage.
